# WHO’s Attempt to Navigate Commercial Influence and Conflicts of Interest in Nutrition Programs While Engaging With Non-State Actors: Reflections on WHO Guidance for Nation States Comment on "Towards Preventing and Managing Conflict of Interest in Nutrition Policy? An Analysis of Submissions to a Consultation on a Draft WHO Tool"

**DOI:** 10.34172/ijhpm.2020.162

**Published:** 2020-09-23

**Authors:** Marc A. Rodwin

**Affiliations:** Law School, Suffolk University, Boston, MA, USA.

**Keywords:** Public Private Partnerships, Ethics, Corruption, Influence

## Abstract

This commentary situates the comments submitted in response to the World Health Organization (WHO) draft guidance on conflicts of interest in national nutrition programs in light of: (1) WHO policies to protect WHO integrity; (2) the Framework of Engagement with Non-State Actors (FENSA); (3) WHO’s attempt to seek funds due to cuts in member contributions; and (4) attempts—often by corporate entities—to redefine conflicts of interest to avoid oversight of conflicts of interest and increase corporate influence. The WHO guidance defines conflicts of interest in ways that deviate from standard legal usage which confuses its analysis and facilitates the creation of conflicted public-private partnerships. The guidance suggests that nations can allow engagement with non-state actors when the benefits are greater than risks without separate check due to conflicts of interest. Instead, the WHO should have recommended that nations seek alternative ways to achieve their goals when non-state actors have significant institutional conflicts of interest.

For many years the World Health Organization (WHO) policy held that it should not establish official relations with non-governmental organizations (NGOs) unless their aims were “in conformity with … principles of the WHO Constitution” and “free from concerns which are … of a commercial or profit-making nature.”^1^ Informal contacts were allowed without the prerogatives of formal status.^1^ WHO maintained formal relations with approximately 200 organizations, predominantly professional, public interest advocate, and humanitarian aid organizations, alongside some industry-financed NGOs. Business actors resisted efforts to distinguish between business interest NGOs and public interest NGOs, and between cooperation with business as opposed to official relations with public-interest associations. Corporations employed business interest NGOs to influence policy and sought representation in United Nations (UN) agencies as so-called partners or stakeholders.^2^ Corporations have tried to influence policy through funding private associations, and non-transparent associations,^3^ and lobbying internationally, just as they have on domestic nutrition policy.^4^

In the 1980s, when nation states decided to freeze or failed to pay their assessed contributions, WHO sought voluntary contributions from national governments for their designated projects. It also later obtained funds from the Bill and Melinda Gates Foundation.^5,6^ Both funding changes reduced WHO’s policy discretion, shifted organizational priorities, and created conflicts of interest.^7-11^ This continued unstable funding spurred WHO to seek alternative funds. By 2010, WHO’s leadership advocated seeking funds from for-profit corporations.

In reaction to criticism, WHO developed guidelines regarding interactions with private sector entities in 2000,^12^ and a policy on Global Health Partnerships in 2010.^13,14^ The 1987 NGO principles and the private sector guidelines from 2000 were replaced in 2016 by the Framework of Engagement with Non-State Actors (FENSA),^15,16^ which allows official relations with non-state actors (namely: NGOs; private sector entities including international business associations; philanthropic foundations; and academic institutions).^17^ For-profit firms eagerly joined public-private partnerships, such as the Global Alliance on Nutrition and Scaling Up Nutrition (SUN) initiative,^18^ while studies revealed that their joining would create conflicts of interest and compromise public policy.^2,19-23^

Two WHO policies provide context for understanding the recent WHO conflict of interest (COI) nutrition program guidance. First, in 1981, the World Health Assembly adopted an international code to restrict marketing of breast-milk substitutes and protect breastfeeding.^24^ Enforcing this policy proved difficult,^25,26^ but it remains a model for WHO public health regulation of harmful commercial practices.^27^ Second, in 2003, the WHO prohibited any engagement with firms that manufacture or market tobacco.^28,29^ The treaty states “Engagement with the tobacco industry is contrary to the UN system’s objectives, fundamental principles and values.”^28^ These policies demonstrate WHO’s ability to regulate private firms to promote public health and restrict engagement with commercial entities.

Critics of the initial proposed framework noted that FENSA’s use of the term *non-state actors* put business-based and public-interest-based actors on an equal footing. Furthermore, it legitimized for-profit firms’ (and affiliated not-for-profit trade associations’) participation in WHO policy development, failed to adequately control conflicts of interest frequently arising from engagement with commercial firms, and did not employ the standard definition of COI used in the law and dictionaries, thereby confusing analysis and undercutting effective responses. Revisions of the proposed framework *did not* remedy these defects, which compromise WHO’s independence and integrity.^15,30-32^

Today, FENSA requires that engagement with non-state actors demonstrate clear public health benefits,^33^ “protect WHO from any undue influence…[regarding] policies, norms and standards;” “not compromise WHO’s integrity, independence, credibility and reputation;” and “be effectively managed, including by, where possible, avoiding COI….” Nevertheless, FENSA permits WHO to have relations with private sector entities that often create conflicts of interest. Furthermore, FENSA creates a flawed process to manage conflicts of interest that result from such engagement.^33^ Critics charge that FENSA facilitates initiatives that undermine addressing childhood obesity and other public health problems.^34^

In 2015, WHO held a technical consultation “on addressing and managing conflicts of interest in the planning and delivery of nutrition programs at country level.”^35^ Subsequently, WHO proposed a “draft approach”^36^ which included a *Report of the Director General*,^37^ an *Introductory Paper*,^38^ and a *Decision-Making Process and *Tool, accompanied by feedback from participants. The *Tool *is a six-step process that governments were encouraged to employ to determine whether to engage with non-state actors, and if they do, to identify and manage conflicts of interest. I refer to these as the draft “conflict of interest nutrition program guidance” or draft “guidance.”

 WHO’s COI guidance says that governments should analyze the purpose of proposed engagement, the risk of planned activities, and the non-state actor’s risk profile. Governments should weigh the risks and benefits of proposed engagement for public health and their reputation and proceed only if the benefits outweigh the risks. If a government engages with non-state actors, it should monitor and manage their conflicts of interest.

The guidance suffers from several defects. First, it lacks clear standards that preclude engagement, or that require its termination, when state, or non-state actors, have significant conflicts of interest. Instead, the guidance recommends that governments perform cost-benefit analysis to decide whether the risks of engagement are worth the benefits. Under the guidance, conflicts of interest never preclude engagement unless all risks exceed all benefits: they are only one factor in the equation. Consequently, nutrition policies will often be compromised by conflicts of interest. This policy departs from the Policy on WHO Engagement with Global Health Partnership, under which, following a favorable cost-benefit assessment of a proposed partnership, the presence of conflicts of interest can still preclude entering the partnership.^13^

In contrast to WHO’s draft nutrition program guidance, effective conflicts-of-interest policy typically prohibits the presence of certain conflicts of interest and sets rules that restrict, or oversee all others.^39-43^ WHO ought to have advised governments to actively seek, and usually employ, alternative ways to reach their goals, rather than engagement which creates significant conflicts of interest. There should be a presumption against engagement with non-state actors with significant conflicts of interest, while allowing rebutting this presumption on limited grounds (not merely when potential benefits are greater than potential risks).^21^

Second, as noted in the 2015 Technical Consultation Report,^35^ the guidance definition of conflicts of interest deviates from the traditional usage in law, regulations, and public administration.^42-44^ The WHO definition confuses analysis and weakens standards to preclude or manage conflicts of interest. I will say more on definitions later.

Third, the guidance suggests that potential engagement should be evaluated based on whether it advances public health goals and maintains program integrity. This overlooks the effect of COI. For instance, manufacturers of vitamin supplements and fortified foods claim to share the government’s goal of reducing population-wide nutritional deficiencies. However, manufacturers want to prioritize reducing vitamin deficiency over other nutritional problems and to reduce deficiencies by promoting their products, while governments should aim at improving nutrition and diets more broadly and promote healthy foods, not merely vitamin-enriched products. The focus on sharing one goal neglects divergence on other goals.^21,45^ Similarly, manufacturers of sugary sodas have sought partnerships to promote exercise as a way to reduce weight gain rather than polices that discourage consumption of soda.^19,21,46,47^

Ralston et al^37^ analyzed 55 comments^48^ submitted by private sector entities, NGOs, member states, UN agencies, and academic institutions in response to the draft nutrition program guidance. They found competing views regarding the risks and benefits of partnerships with non-state actors. For-profit firms, their not-for-profit trade associations, and the United States wanted to promote such partnership, and believed that policies control conflicts of interest created obstacles. In contrast, most NGOs, some academic institutions, and most governments were wary of engagement due to conflicts of interest. The comments, Ralston et al say, reflect competing policy frames: (1) a “collaboration and partnership fame,” which favored such partnerships and (2) a “conflict and restricted engagement frame,” which disfavors such partnership because of “tensions between public health and food industry interests.”^48^

Ralston et al vacillate when they assess these competing policy frames. They write that “commercial sector actors strategically use frames as ‘weapons of advocacy’ to promote … their economic and political interests.”^48^ However, other times they suggest that these two policy frames have comparable support. They write that there are “high levels of contestation surrounding the very concept of COI,”^48^ and that the “literature has recognized the ambiguity and malleability of COI.”^48^

In fact, the legal concept, *COI*, has a long-standing, commonly understood core meaning, even though some writers have tried to redefine the concept.^46^ A COI arises whenever activities or relationships compromise the loyalty or independent judgment of an individual or institution who is obligated to serve a party or perform certain roles. Multiple interests often pull individuals in different directions, but only when they compromise fulfilling *obligations* is there a COI. There are two broad types of conflicts of interest: (1) conflicts between an individual’s obligations and their financial or other self-interest; (2) conflicts resulting from an individual’s divided loyalties, dual roles or conflicting duties, sometimes referred to as conflicts of commitment.^49^ The two types of conflict reflect different sorts of problems but are not mutually exclusive. Most dictionaries distinguish between financial and dual loyalty conflicts,^50^ but some do not.^51^ Legal texts, treatises, and organizational policies typically employ the term in ways consistent with such definitions.

In recent years, some authors have attempted to redefine conflicts of interest to include so-called* intellectual* or *non-financial* conflicts.^44^ Some writers would bar individuals from engaging in activities because of their prior work or intellectual commitments, just as the law has traditionally barred individuals from undertaking activities due to financial conflicts of interest.^52^ Others argue that we should not exclude individuals or organizations with financial conflicts of interest because we do not bar individuals with so-called *intellectual conflicts*.^53^ These new definitions are not compatible with the traditional legal approach. Certainly, all sorts of biases can compromise individual judgment, but not all biases are conflicts of interest, and other biases should be addressed separately.

The industry-affiliated groups that commented on the nutrition program guidance often defined COI in ways that undermine their effective oversight or confuse conflicts of interest with other sources of bias or differing world-views. For example, the Alliance for Food & Health suggests that political, religious, philosophical biases are on par with financial conflicts of interest.^54^ The Global Dairy Platform says conflicts of interest are not only financial and include “statements in publications..., and other biases.”^55^

In general, industry-affiliated entities sought a role in developing and implementing polices, even when their interests and activities run counter to public policies. The Grocery Manufacturers Association says that “agreement with specific policies…should not be a prerequisite for engagement.”^56^ And the SUN Movement Secretariat, which promotes public-private partnerships, says that addressing “conflicts of interest should initially start from a positive perspective, not from negative assumptions,”^57^ thereby ignoring that conflicts of interest represent a risk to be controlled. SUN’s Secretariat also says that “tools to manage conflicts of interest should serve as a mechanism to enable, rather than prevent partnerships.”^57^ But in fact, managing conflicts of interest typically requires restricting partnerships and activities that create conflicts of interest. In contrast to commercial firms and their supporters, various NGOs and some ethics and policy scholars clearly articulated what constitutes a COI and proposed guidance changes to promote their effective oversight.^36,58-65^

Today, policy-makers recognize that conflicts of interest compromise public policy. Too often, however, they do little more than require that individuals disclose their financial interests, a step necessary for managers to identify conflicts of interest, but insufficient to control them.^41,42,66^ An effective response requires changing financial relationships or activities, and will preclude initiatives that create institutional conflicts of interest and also exclude individuals who have conflicts of interest.^40,67^ Governments and public health actors considering collaboration regarding nutrition with non-state actors, such as for-profit firms and foundations, need to do much more than conduct a cost-benefit assessment of the risks of such collaboration. They need policies that generally preclude so-called partnerships with stakeholders and other entities compromised by institutional conflicts of interest, as well as screening of conflicted individual actors, or, careful management of their conduct.

## Acknowledgements

 Judith Richter and Jonathan Marks provided helpful comments on a draft. Thanks to Kyla J. Goolsby for research assistance.

## Ethical issues

 Not applicable.

## Competing interests

 MAR was invited by the WHO as an expert on conflicts of interest to participate in the 2015 WHO technical consultation “Addressing and managing conflicts of interest in the planning and delivery of nutrition programmes at country level.”

## Author’s contribution

 MAR is the single author of the paper.
